# The Longitudinal Relationship Between eHealth Literacy, Health-Promoting Lifestyles, and Health-Related Quality of Life Among College Students: A Cross-Lagged Analysis

**DOI:** 10.3389/fpubh.2022.868279

**Published:** 2022-07-08

**Authors:** Shaojie Li, Guanghui Cui, Feixiang Zhou, Siyue Liu, Yicong Guo, Yongtian Yin, Huilan Xu

**Affiliations:** ^1^Shandong University of Traditional Chinese Medicine, Jinan, China; ^2^Xiangya School of Public Health, Central South University, Changsha, China

**Keywords:** eHealth literacy, health-related quality of life, health-promoting lifestyles, cross-lagged panel analysis, longitudinal study, college students

## Abstract

**Objectives:**

This study aimed to explore the longitudinal associations between eHealth literacy, health-promoting lifestyles, and health-related quality of life (HRQoL) among college students.

**Methods:**

From December 2019 (T1) to December 2020 (T2), we administered the eHealth literacy scale, Short-Form Health Survey (SF-12), and Short-Form Health-Promoting Lifestyle Profile Scale to 1,181 college students in Jinan, China. Participants were recruited for 12 months for the two-stage survey.

**Results:**

Stable positive correlations were shown between eHealth literacy, health-promoting lifestyles, and HRQoL across time. The cross-lagged analysis showed that eHealth literacy at T1 predicted health-promoting lifestyles at T2 (β = 0.080, *P* = 0.006); however, health-promoting lifestyles at T1 did not predict eHealth literacy at T2 (β = −0.026, *P* = 0.499). HRQoL at T1 predicted health-promoting lifestyles at T2 (β = 0.147, *P* < 0.001); however, similar to the eHealth literacy finding, health-promoting lifestyles at T1 did not predict HRQoL at T2 (β = 0.045, *P* = 0.142). eHealth literacy was also bi-directionally associated with HRQoL, and the prediction effect of eHealth literacy at T1 to HRQoL at T2 (β = 0.078, *P* = 0.008) was slightly higher than the prediction effect of HRQoL at T1 to eHealth literacy at T2 (β = 0.074, *P* = 0.023).

**Conclusion:**

eHealth literacy and HRQoL may be antecedents for college students' health-promoting lifestyles. There may be significant bi-directional relationships between eHealth literacy and HRQoL.

## Introduction

According to the China Internet Network Information Center, as of March 2020, there were 904 million Internet users in China, of whom student Internet users accounted for the highest proportion, at 26.9% (1). As active Internet users, college students tend to search the Internet for health information (2, 3). However, diverse health information sources and content, as well as the imbalanced quality of online health information may hinder students' efforts to obtain, evaluate, and use accurate health information (4). eHealth literacy may help solve this problem. eHealth literacy refers to individuals' ability to collect and evaluate online health information through electronic media and to use this information to address their own health problems (5). Studies both prior to and during the COVID-19 pandemic all suggest that individuals with higher eHealth literacy were more able to find reliable online health information resources (6, 7). Therefore, developing college students' eHealth literacy is essential for them to effectively and accurately use Internet health information to promote their health.

Attending university is an important stage for young adults transitioning from school to society and is an important time for establishing positive health behaviors. However, college students often have unhealthy lifestyles, resulting in an increased risk of adverse health outcomes. A survey involving 2,422 Chinese college students find that the prevalence of lack of physical activity, sleep disorder, and bad eating habits were 62.0, 42.6, and 29.8%, respectively (8). These unhealthy lifestyles were all associated with an increased risk of health problems such as depression, anxiety, and general poor health-related quality of life (HRQoL) (8–10). It is, therefore, necessary to determine the factors that are related to a healthy lifestyle and HRQoL among college students; this question also has important public health significance for facilitating students' present and future health.

Health literacy is a recognized factor that influences individuals' health behavior and outcomes (11). As an extension of general health literacy, eHealth literacy may also be related to healthy behaviors and health outcomes. Previous studies find that college students with high eHealth literacy are more likely to adopt healthy lifestyles (12–14); this finding has also been confirmed among older adults (15), nurses (16), and adult Internet users (17). These studies provide preliminary evidence that eHealth literacy is closely related to healthy lifestyles. Studies have also explored the underlying mechanisms of the association between eHealth literacy and healthy lifestyles among nursing students. Specifically, they find that eHealth literacy has an indirect effect on healthy lifestyles through social media use to obtain health information, online health information-seeking behaviors, and self-care agency (18). It should be noted that the above studies were all cross-sectional; therefore, they fail to clarify the direction of effects between eHealth literacy and healthy lifestyles. As is well-known, cross-sectional studies analyze population data at a single point in time and are subject to limitations, such as the inability to make causal inferences and difficultly in explaining the direction of associations (19). Therefore, it is necessary to further explore the longitudinal association between the factors of eHealth literacy and healthy behaviors, and to clarify the direction of their effects. The transactional model of eHealth literacy suggests that such literacy can promote informing and engaging patients, which leads to an increase in the frequency of information-seeking, more effective patient-provider communication, more proactive health behaviors, and better HRQoL (20). Moreover, the model suggests that individuals' health behaviors and HRQoL will, in turn, affect their motivation to use eHealth services, thereby indirectly affecting their eHealth literacy (20). This suggests that eHealth literacy may be bi-directionally associated with healthy lifestyles. However, the longitudinal association between these factors has not been explored to date.

The relationship between eHealth literacy and health status has recently received widespread attention. Studies show that low eHealth literacy can affect various health aspects, such as self-care for chronic diseases (21), depression, insomnia, post-traumatic stress disorder (22), and cognitive health (15). However, few studies focus on the relationship between eHealth literacy and comprehensive health measures (such as HRQoL), especially among college students. As an indicator of comprehensive health status, HRQoL reflects individuals' perceptions of their physical and mental health and living status within their current culture, value system, and related environment (23). A recent study shows that eHealth literacy helped protect patients' HRQoL from the negative effects of fear associated with COVID-19 during the pandemic (24). In addition, a previous study of patients with chronic obstructive pulmonary disease finds that eHealth literacy was positively correlated with HRQoL (25); a similar result was reported for older adults (26). However, it is not known whether these findings can be generalized to a college student population. Previous studies find that college students with high eHealth literacy used health service resources more effectively to improve their health status (27). Moreover, eHealth literacy has been found to be closely related to college students' mental health (28). As HRQoL comprises both physical and mental health, the eHealth literacy of college students may be related to their HRQoL. However, there is currently a lack of evidence in this area. Similarly, to the best of our knowledge, no previous research has explored the longitudinal relationships between eHealth literacy and HRQoL. A previous study finds that Chinese medical students with good self-reported health were more likely to have a high level of eHealth literacy (29). This may imply that HRQoL, in turn, affects eHealth literacy. However, based on existing research, it is unknown whether eHealth literacy has a prospective impact on college students' HRQoL or vice versa.

Previous cross-sectional studies confirm that health-promoting lifestyles are closely related to HRQoL (26, 30, 31); however, similar to the aforementioned research, the longitudinal relationship between them was not examined. The nature of cross-sectional studies limits understanding of the temporal relationship and interaction between health-promoting lifestyles and HRQoL. To address this limitation, longitudinal research is necessary. Previous randomized controlled trials show that health-promoting lifestyle counseling and education improved HRQoL among women (32, 33). The health promotion model asserts that individuals can promote health through behavioral changes to achieve positive health outcomes (34). Although this suggests that health-promoting lifestyles may be an antecedent of HRQoL, this hypothesis lacks the support of longitudinal observational studies. A cohort study finds that healthy lifestyles (in terms of physical activity, alcohol consumption, cigarette smoking, and so forth) predicted HRQoL in people with multiple sclerosis (35). However, the study did not explore the reverse relationship—whether participants' baseline HRQoL would affect their healthy lifestyles. Further, it is not known whether the results obtained from patients with multiple sclerosis can be generalized to healthy college students. Given the sociodemographic and health differences between college students, patients, and women in general, the findings of these prior studies also may not be generalizable to college students. In addition, it is unclear whether HRQoL predicts engagement in health-promoting lifestyles. A previous 5-year follow-up survey of young people aged 26–36 years old revealed a bi-directional relationship between healthy lifestyles and mood disorders (36). Given that mood health is one of the important aspects of HRQoL (37), one may infer that health-promoting lifestyles are also bi-directionally related to HRQoL. Therefore, a longitudinal research design must be adopted to further explore the relationship between college students' health-promoting lifestyles and HRQoL.

In view of the limitations and research gaps in previous studies, we conducted a 1-year follow-up study to analyze the longitudinal relationship between eHealth literacy, health-promoting lifestyles, and HRQoL among college students; to clarify the directions of effects among the three factors; and to provide high quality scientific evidence to help derive relevant interventions in the future.

## Materials and Methods

### Participants

This is a 1-year longitudinal study of 1,181 college students in China. In this study, we followed the Strengthening the Reporting of Observational Studies in Epidemiology (STROBE) Statemen (38). The required sample size was calculated using the formula $n=\frac{{\mu_{\alpha /2}}^{2}\pi \left(1-\pi \right)}{\delta^{2}}$. Previous studies show that 32.6% of college students in Jinan have adequate eHealth literacy (39). Therefore, in this study, π = 0.326, α = 0.05, μ_α_/2 = 1.96, and δ = 0.05, resulting in a required sample size of 794. To control for invalid survey samples, we increased the sample size by 10%, resulting in 873 as the minimum sample size required for this study.

The sample population included all college students in 30 classes of a university in Jinan City, Shandong Province. The inclusion criteria were smartphone ownership and absence of any clinical diagnosis of major physical and psychological diseases, such as cancer, COVID-19, depression, anxiety, and so forth, based on the participants' self-report. College students with a clinically diagnosed disease were not considered because this study focused on non-clinical samples in an attempt to provide scientific evidence for early prevention and intervention with respect to college students' eHealth literacy, health-promoting lifestyles, and HRQoL. Prior to initiating the study, we contacted the university's student administration to obtain their consent, after which we announced the recruitment to the class. During extracurricular time in December 2019 (T1), uniformly trained investigators administered a self-reported questionnaire to 1,235 participants. In December 2020 (T2, 12 months after T1), we followed up with the same participants. Each survey took ~30 min. Preset codes were used to match participant responses for the T1 and T2 measurements, which safeguarded participants' identity. By matching the preset codes, we found that 54 people could not be followed up with, because they were absent from school during the T2's survey; these participants' data were excluded from the final sample. Finally, 1,181 participants who provided data twice were included in the study. All participants were gathered in a classroom to complete the survey, and all provided informed consent prior to the first survey and agreed to participate in the follow-up survey. The study was approved by the Medical Ethics Committee of the Second Affiliated Hospital of Shandong University of Traditional Chinese Medicine (identification code: 2019-03-11). All research procedures adhered to the guidelines stipulated in the Declaration of Helsinki.

### Measurements

#### eHealth Literacy

Participants' eHealth literacy was measured using the Chinese version of the eHealth Literacy Scale (eHEALS), developed by Norman (40) and translated by Yu et al. (41). The scale comprises eight items, each rated on a scale from 1 (disagree) to 5 (agree), with a total score ranging from 8 to 40 and higher scores indicating higher eHealth literacy. The Cronbach's α coefficient of the scale in this study was 0.937 and 0.965 at T1 and T2, respectively.

#### Health-Promoting Lifestyles

Health-promoting lifestyles were measured using the short-form health-promoting lifestyle profile (HPLP), which was revised by Wei et al. (42) from the 48-item HPLP (43). The 24-item scale includes self-actualization, health responsibility, exercise, nutrition, interpersonal support, and stress management (with four items each) scored on a four-point Likert scale ranging from 1 (never) to 4 (always); higher scores indicate higher health-promoting lifestyles. The Cronbach's α coefficient of the scale in this study was 0.922 and 0.940 at T1 and T2, respectively.

#### HRQoL

HRQoL was measured using the Chinese version of the Short-Form Health Survey (SF-12) (44). The scale has 12 items that cover eight dimensions: physical functioning (PF), role physical (RP), role emotional (RE), bodily pain (BP), general health (GH), vitality (VT), social functioning (SF), and mental health (MH). The eight dimensions are divided into two summaries: physical component summary (including PF, RP, BP, and GH) and mental component summary (including VT, SF, RE, and MH) (45). We used the converted standard total score (according to the scoring standard) (46) to evaluate individuals' HRQoL, where higher scores indicated higher HRQoL. The Cronbach's α coefficient of the scale in this study was 0.791 and 0.784 at T1 and T2, respectively.

#### Sociodemographic Variables

Covariates consisted of participants' age, sex, Chinese ethnic group, place of residence, academic major, and self-reported family economic level.

### Statistical Methods

IBM SPSS Statistics, version 23 (IBM SPSS Statistics, Armonk, NY, USA) was used to conduct descriptive and correlation analyses. Data were presented as *n* (%) for categorical variables and mean (standard deviation, SD) for numeric variables. *Z*-test was used to analyze the differences in the mean scores of eHealth literacy, health-promoting lifestyles, and HRQoL between the 54 participants with missing follow-up data and the 1,181 participants in the two surveys. Pearson correlation coefficients were used to test the association between eHealth literacy, health-promoting lifestyles, and HRQoL. IBM SPSS AMOS 23.0 was used to perform structural equation modeling with maximum likelihood to test a cross-lagged model. This model considers two or more variables at different time points, in which the estimated path coefficients of the pre-measured variables affect the post-measured variables. That is, the variables have time sequence relationship, which conforms to the principle of epidemiological causal inference, and can be used to explore the mutual predictive relationships or quasi-causal relationships between variables (47). Specifically, in the model, the autoregressive coefficients of the same variable measured twice test the stability of the variables over time, and the regression coefficients of the pre-test and *post-test* of different variables test the mutual prediction effect between variables. In this study, we constructed two 3 × 3 cross-lagged models (that is, from T1 eHealth literacy, health-promoting lifestyles, and HRQoL to T2 eHealth literacy, health-promoting lifestyles, and HRQoL, for a total of nine regression coefficients) to test the mutual predictive effects of the three variables. Model 1 was unadjusted, while Model 2 was adjusted for age, sex, Chinese ethnic group, residence, academic major, and self-reported family economic level. By comparing the magnitude and significance of these standardized regression coefficients, the strength and direction of the associations between variables were determined (47). The chi-squared value (χ^2^) divided by the degrees of freedom (df) of < 5,000, a comparative fit index (CFI) and Tucker-Lewis index (TLI) >0.900, and a root mean square error approximation (RMSEA) < 0.080 (48), indicated an acceptable cross-lagged model fit.

## Results

### Descriptive Statistics

Table 1 shows the characteristics of the demographic variables at T1 and the means and standard deviations for the eHEALS score of eHealth literacy, the HPLP score of health-promoting lifestyles, and the SF-12 score of HRQoL. The *Z*-test results showed that at the time of the first survey, there were no statistically significant differences in the scores of 54 participants with missing follow-up data and the 1,181 participants who completed two surveys on eHEALS score, HPLP score, and SF-12 score; this indicates that the missing samples during follow-up have no impact on the study.

**Table 1:** Descriptive statistic for all variables (*N* = 1,181).

**Variables**	
Age, mean (SD)	18.91 (0.85)
**Sex**, ***n*** **(%)**	
Man	582 (49.3)
Woman	599 (50.7)
**Chinese ethnic groups**, ***n*** **(%)**	
Han nationality	1,138 (96.4)
Other Chinese minority nationalities	43 (3.6)
**Residence**, ***n*** **(%)**	
Urban	518 (43.9)
Rural	663 (56.1)
**Academic major**, ***n*** **(%)**	
Medicine	510 (43.2)
Others	671 (56.8)
**Family economic level**, ***n*** **(%)**	
High	190 (16.1)
Medium	827 (70.0)
Low	164 (13.9)
eHEALS scores T1, mean (SD)	29.50 (6.91)
eHEALS scores T2, mean (SD)	29.04 (8.36)
HPLP scores T1, mean (SD)	65.84 (11.68)
HPLP scores T2, mean (SD)	68.17 (13.33)
SF-12 scores T1, mean (SD)	75.99 (13.67)
SF-12 scores T2, mean (SD)	77.92 (14.70)

### Relationship Between eHealth Literacy, Health-Promoting Lifestyles, and HRQoL

The Pearson's correlation analysis showed that eHealth literacy was related to health-promoting lifestyles and HRQoL at two time points, and that health-promoting lifestyles were also related to HRQoL at two time points (Table 2).

**Table 2:** Correlation matrix (Pearson r and two-tailed *P*-value ^a^) of eHealth literacy, health-promoting lifestyles and HRQoL at two time-points.

**Variables**	**eHealth literacy T1**	**eHealth literacy T2**	**Health-promoting lifestyles T1**	**Health-promoting lifestyles T2**	**HRQoL T1**	**HRQoL T2**
**eHealth literacy T1**						
*r*	1	0.262	0.335	0.232	0.190	0.177
*P-*value	—^b^	<0.001	<0.001	<0.001	<0.001	<0.001
**eHealth literacy T2**						
*R*	0.262		0.086	0.172	0.119	0.190
*P-*value	<0.001	—	0.003	<0.001	<0.001	<0.001
**Health-promoting lifestyles T1**						
*r*	0.335	0.086	1	0.479	0.420	0.253
*P-*value	<0.001	0.003	—	<0.001	<0.001	<0.001
**Health-promoting lifestyles T2**						
*r*	0.232	0.172	0.479	1	0.316	0.471
*P-*value	<0.001	<0.001	<0.001	—	<0.001	<0.001
**HRQoL T1**						
*r*	0.190	0.119	0.420	0.316	1	0.492
*P-*value	<0.001	<0.001	<0.001	<0.001	—	<0.001
**HRQoL T2**						
*r*	0.177	0.190	0.253	0.471	0.492	1
*P-*value	<0.001	<0.001	<0.001	<0.001	<0.001	—

^a^* For all associations, the correlation is significant at a level of .05 (two-tailed). ^b^ Not applicable*.

### Cross-Lagged Analysis

**Figure 1:**
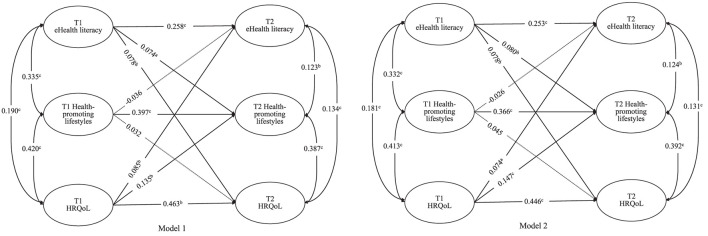
Cross-lagged analysis of eHealth literacy, health-promoting lifestyles and HRQoL at two time points. Model 1 was unadjusted models. Model 2 adjusted for age, sex, Chinese ethnic groups residence and academic major, and self-reported family economic level, the standardized coefficients of the control variables were not displayed in the model but were included in the analyses. ^a^ *P* < 0.05, ^b^ *P* < 0.01, ^c^ *P* < 0.001; All coefficients are standardized coefficients; The solid line represents the path that is statistically significant (*P* < 0.05), and the dashed line represents the path that is not statistically significant (*P* > 0.05).

Figure 1 shows that Model 1 and Model 2 achieved consistent results; that is, except for “health-promoting lifestyles T1 to eHealth literacy T2” and “health-promoting lifestyles T1 to HRQoL T2,” other paths were all statistically significant. As the significance of each coefficient did not change between the unadjusted and adjusted models, we did not conduct subgroup analyses.

Specifically, the autoregressive coefficients of the three variables from T1 to T2 were all statistically significant in both the unadjusted model and adjusted models, with an acceptable model fit (χ^2^/*df* = 2.680, CFI = 0.983, TLI = 0.943, RMSEA = 0.038). After adjusting for sociodemographic variables, Model 2 shows that eHealth literacy at T1 predicted health-promoting lifestyles at T2 (β = 0.080, *P* = 0.006); however, health-promoting lifestyles at T1 did not predict eHealth literacy at T2 (β = −0.026, *P* = 0.499). HRQoL at T1 predicted health-promoting lifestyles at T2 (β = 0.147, *P* < 0.001); however, similar to the eHealth literacy finding, health-promoting lifestyles at T1 did not predict HRQoL at T2 (β = 0.045, *P* = 0.142). In addition, eHealth literacy was bi-directionally associated with HRQoL, and the prediction effect of eHealth literacy at T1 to HRQoL at T2 (β = 0.078, *P* = 0.008) was slightly higher than the prediction effect of HRQoL at T1 to eHealth literacy at T2 (β = 0.074, *P* = 0.023). Details are shown in Table 3.

**Table 3:** Bootstrapped estimation of each path of the cross-lagged model.

**Path**	**Model 1 (unadjusted model)**				**Model 2 (adjusted model)**			
	**β**	**SE**	**95% CI**	***P*-value**	**β**	**SE**	**95% CI**	***P*-value**
eHealth literacy T1 to eHealth literacy T2	0.258	0.035	0.190–0.326	<0.001	0.253	0.035	0.184–0.322	<0.001
Health-promoting lifestyles T1 to health-promoting lifestyles T2	0.397	0.031	0.338–0.458	<0.001	0.366	0.031	0.303–0.427	<0.001
HRQoL T1 to HRQoL T2	0.463	0.029	0.404–0.517	0.001	0.446	0.029	0.387–0.500	<0.001
eHealth literacy T1 to health-promoting lifestyles T2	0.074	0.030	0.013–0.132	0.018	0.080	0.029	0.023–0.134	0.006
eHealth literacy T1 to HRQoL T2	0.078	0.030	0.020–0.138	0.008	0.078	0.030	0.020–0.138	0.008
Health-promoting lifestyles T1 to eHealth literacy T2	−0.036	0.037	−0.108–0.037	0.334	−0.026	0.038	−0.099–0.051	0.499
Health-promoting lifestyles T1 to HRQoL T2	0.032	0.030	−0.024–0.093	0.251	0.045	0.031	−0.015–0.107	0.142
HRQoL T1 to eHealth literacy T2	0.085	0.032	0.024–0.149	0.006	0.074	0.033	0.010–0.142	0.023
HRQoL T1 to health-promoting lifestyles T2	0.1325	0.029	0.074–0.190	0.001	0.147	0.028	0.091–0.201	<0.001

*β, standardized regression coefficient; SE, standardized error; CI, confidence interval (bias-corrected bootstrap confidence intervals)*.

## Discussion

To the best of our knowledge, this is the first study to analyze the longitudinal associations among eHealth literacy, health-promoting lifestyles, and HRQoL. Our results reveal the directionality of relationships among eHealth literacy, health-promoting lifestyles, and HRQoL among Chinese college students, and provide a reference for future related intervention research. We found that after adjusting for age, sex, Chinese ethnic groups, academic major, and self-reported family economic level, early eHealth literacy and HRQoL were predictors of health-promoting lifestyles 1 year later; however, this relationship was not bi-directional. There were bi-directional relationships between eHealth literacy and HRQoL.

In this study, the autoregressive coefficients in the cross-lagged model were all significant, indicating that eHealth literacy, health-promoting lifestyles, and HRQoL were all stable across time. One important finding was that eHealth literacy predicted health-promoting lifestyles, which expands previous cross-sectional study findings by introducing a longitudinal focus (12). Previous longitudinal studies show that higher health literacy promotes individual physical exercise (49) and colorectal cancer screening (50), and helps individuals manage their health better (51). As eHealth literacy is an extension of health literacy, these studies also indirectly reflect a possible longitudinal relationship between eHealth literacy and healthy lifestyles. According to the health literacy skill model (52), eHealth literacy is a health literacy skill, which can theoretically affect individual health behavior and outcomes. Previous studies show that individuals with higher eHealth literacy generally have higher health information-seeking behaviors (53, 54). This suggests that higher eHealth literacy among college students may lead to a stronger belief in health maintenance, making them more likely to actively maintain and promote their health. Thus, college students with high eHealth literacy engage in lifestyles that promote health. However, our study also found that health-promoting lifestyles at T1 did not affect eHealth literacy at T2; this departs from the cognitive-behavioral perspective that behavior strengthens cognition (55). However, we must consider that eHealth literacy differs from health literacy, as the former does not reflect individuals' health knowledge reserves or cognition; it does, however, emphasize individuals' ability to obtain health information and related medical care services and to make decisions based on electronic media. Engaging in healthy lifestyles, such as getting physical exercise and optimal fruit and vegetable intake, might not improve individuals' ability to retrieve and evaluate electronic health information. Rather, changes in eHealth literacy are related to socioeconomic status, knowledge reserve, and continuous practice (56–59). College students who adopt health-promoting lifestyles may nevertheless spend their time and energy on electronic media entertainment and social interactions, yet have little understanding of electronic health information on websites and other platforms, or may not be able to differentiate high quality from poor quality electronic health information. This may be why health-promoting lifestyles do not affect eHealth literacy. As literature on the longitudinal relationships between eHealth literacy and healthy lifestyles is limited, our results and interpretations require further exploration.

Our study also found a bi-directional relationship between eHealth literacy and HRQoL among college students; that is, eHealth literacy at T1 predicted HRQoL at T2, and in turn, HRQoL at T1 also predicted eHealth literacy at T2. This finding extends the association between eHealth literacy and HRQoL to the college student population and this is the first study to determine the direction of the effect. It is important to note, however, that a cross-lagged model was used to identify longitudinal and directional associations among eHealth literacy and HRQoL, not causal relationships. Given the relatively small coefficients in the model, results must be interpreted with caution. First, as for the effect of eHealth literacy at T1 on HRQoL at T2, this may be explained from the perspective of the three core abilities, according to the definition of eHealth literacy: the collection, evaluation, and application of online health information. The ability to collect online health information reflects, to a certain extent, individuals' health responsibility awareness and online health information-seeking behavior, whereby higher collection ability is associated with a stronger initiative to collect health information and a better ability to find suitable solutions to personal health conditions. Similarly, individuals with a better ability to evaluate health information may have richer health knowledge reserves, allowing them to differentiate between high and low-quality health information. Furthermore, the ability to apply health information involves transforming online health information into health behaviors and health-related decisions, whereby stronger application ability facilitates transforming online health information into practical actions for maintaining and promoting personal health. Therefore, individuals with higher eHealth literacy experience better HRQoL outcomes. Second, in terms of the effect of HRQoL at T1 on eHealth literacy at T2, it may be related to individuals with high HRQoL being more likely to access health services. Previous study also finds that differing degrees of individual exposure to eHealth services led to a wide range of eHealth literacy among individuals (60). This suggests that college students with higher HRQoL may pay closer attention to eHealth services out of concern over their own health conditions, which indirectly improves their eHealth literacy. A Taiwanese study finds that college students with higher health status had higher eHealth literacy (61). These studies provide support for the bi-directional association between eHealth literacy and HRQoL.

Similar to previous cross-sectional studies (31), we also found that health-promoting lifestyles were significantly positively correlated with HRQoL at two time points. It should be noted that most previous studies regard health-promoting lifestyles as an antecedent of HRQoL. However, our cross-lagged model showed that early health-promoting lifestyles did not significantly predict follow-up HRQoL, even though the opposite path was significant. This may be because, compared with patients with chronic diseases and older adults, college students' HRQoL was generally high; therefore, changes in HRQoL and health-promoting lifestyles during 12 months may have been subtle. A 3-year longitudinal study of adolescents in Australia found that HRQoL remained stable over time, with little change (62). Therefore, identifying the predictive effect of health-promoting lifestyles on college students' HRQoL may require a longer follow-up period. In addition, predicting follow-up health-promoting lifestyles from early HRQoL may be influenced by the higher self-efficacy seen among college students with high HRQoL (63). Previous studies in South Korea reported that perceived health status and self-efficacy were the most powerful predictors of health-promoting lifestyles among college students (64, 65). According to the health belief model, individuals' perception of health (including perceived benefits and threats) and self-efficacy are important factors in predicting health behaviors (66). This suggests that college students with high HRQoL may have a stronger perception of their health status and self-efficacy for maintaining and promoting health, thus prompting them to adopt healthy lifestyles.

It is worth noting that the 1-year follow-up period of this study happened to include the COVID-19 pandemic; therefore, one might propose that the pandemic may have affected the results of this study. However, given the autoregressive tests, the three variables were highly stable. In addition, the COVID-19 outbreak had not yet occurred at the time of the first survey, and when we conducted the second survey, China had entered the stage of normalization of epidemic prevention and control, college students had already studied collectively in schools for 4 months, and none of the participants had ever been infected with COVID-19. Therefore, we believe that the COVID-19 pandemic did not affect the results of this study.

This study has several limitations. First, this was a 1-year longitudinal study; future studies should extend the follow-up period and collect data at multiple time points, which would help to clarify the long-term relationship and underlying mechanisms between eHealth literacy, health-promoting lifestyles, and HRQoL. Second, our survey collected the sex of the participants at birth only, not their self-perceived gender identity, which may have introduced bias, because previous studies report that gender identity may affect individuals' HRQoL (67). Third, as we mentioned in the method section, the associations obtained by the cross-lagged model were mutually predictive relationships or quasi-causal relationships. Therefore, this study could not clearly determine the causal relationships between eHealth literacy, health-promoting lifestyles, and HRQoL. In the future, causality must be explored through experimental research designs, such as intervention studies. Fourth, as we only selected Chinese college students without disease as participants, the results may not be generalizable to college students with diseases, other populations or countries. Fifth, as this study mainly focuses on the relationship between eHealth literacy, health-promoting lifestyles, and college students' comprehensive health (that HRQoL), we did not analyze the physical health and mental health in SF-12 separately. Considering that physical health and mental health are the two aspects of an individual's health status, the associations of physical and mental health with eHealth literacy and health-promoting lifestyles may differ; this must be further explored in the future. Finally, we used self-report surveys, which may have introduced information bias.

## Conclusions

eHealth literacy and HRQoL may be antecedents of college students' health-promoting lifestyles. There may be significant bi-directional relationships between eHealth literacy and HRQoL.

## Data Availability Statement

The datasets can be made available to any interested person(s) contacting the corresponding author via email.

## Ethics Statement

The studies involving human participants were reviewed and approved by Medical Ethics Committee of the Second Affiliated Hospital of Shandong University of Traditional Chinese Medicine. The patients/participants provided their written informed consent to participate in this study.

## Author Contributions

SLi and GC designed, implemented the entire study, and wrote the first draft. SLi, GC, and YY conducted surveys. FZ, SLiu, and YG contributed to data disposal. YY and HX revised the first draft. All authors approved the final manuscript.

## Conflict of Interest

The authors declare that the research was conducted in the absence of any commercial or financial relationships that could be construed as a potential conflict of interest.

## Publisher's Note

All claims expressed in this article are solely those of the authors and do not necessarily represent those of their affiliated organizations, or those of the publisher, the editors and the reviewers. Any product that may be evaluated in this article, or claim that may be made by its manufacturer, is not guaranteed or endorsed by the publisher.
